# Leveraging artificial intelligence to optimize COVID-19 robust spread and vaccination roll-out strategies in Southern Africa

**DOI:** 10.3389/frai.2022.1013010

**Published:** 2022-10-13

**Authors:** Thuso Mathaha, Mhlambululi Mafu, Onkabetse V. Mabikwa, Joseph Ndenda, Gregory Hillhouse, Bruce Mellado

**Affiliations:** ^1^School of Physics and Institute for Collider Particle Physics, University of the Witwatersrand, Johannesburg, South Africa; ^2^Department of Physics, Case Western Reserve University, Cleveland, OH, United States; ^3^Department of Mathematics and Statistical Sciences, Botswana International University of Science and Technology, Palapye, Botswana; ^4^Department of Physics and Astronomy, Botswana International University of Science and Technology, Palapye, Botswana; ^5^iThemba LABS, National Research Foundation, Somerset West, South Africa

**Keywords:** COVID-19, comorbidities, vaccine rollout, artificial intelligence, Botswana

## Abstract

The outbreak of coronavirus in the year 2019 (COVID-19), caused by severe acute respiratory syndrome coronavirus 2 (SARS-CoV-2) prompted widespread illness, death, and extended economic devastation worldwide. In response, numerous countries, including Botswana and South Africa, instituted various clinical public health (CPH) strategies to mitigate and control the disease. However, the emergence of variants of concern (VOC), vaccine hesitancy, morbidity, inadequate and inequitable vaccine supply, and ineffective vaccine roll-out strategies caused continuous disruption of essential services. Based on Botswana and South Africa hospitalization and mortality data, we studied the impact of age and gender on disease severity. Comparative analysis was performed between the two countries to establish a vaccination strategy that could complement the existing CPH strategies. To optimize the vaccination roll-out strategy, artificial intelligence was used to identify the population groups in need of insufficient vaccines. We found that COVID-19 was associated with several comorbidities. However, hypertension and diabetes were more severe and common in both countries. The elderly population aged ≥60 years had 70% of major COVID-19 comorbidities; thus, they should be prioritized for vaccination. Moreover, we found that the Botswana and South Africa populations had similar COVID-19 mortality rates. Hence, our findings should be extended to the rest of Southern African countries since the population in this region have similar demographic and disease characteristics.

## 1. Introduction

Since its emergence, the novel COVID-19 disease has rapidly spread worldwide, despite various elaborate efforts to contain the infection, resulting in high mortality rates (Dong et al., 2020; Li Q. et al., 2020). Owing to the severity of the COVID-19 disease, the World Health Organization (WHO) has declared it a Public Health Emergency of International Concern because it presents a high risk to countries with vulnerable health systems (Sohrabi et al., 2020; World Health Organization, 2020). As a result, WHO and the US Centers for Disease Control and Prevention (CDC) have issued several non-pharmaceutical preventive measures; for instance, physical distancing and lockdown, quarantine of cases and contacts, handwashing, and mask-wearing effectively reduce COVID-19 case incidence (Fagbule, 2019; Anderson et al., 2020; Jin Y.-H. et al., 2020; Tuite et al., 2020; Du et al., 2022). Currently, there is no specific confirmed COVID-19 antiviral treatment (Rothan and Byrareddy, 2020; Bharati et al., 2022; Rahmah et al., 2022), triggering intense global scientific research and development initiatives to develop safe and effective vaccines that protect from SARS-CoV-2 infections (Ahn et al., 2020; Le et al., 2020). This has resulted in several of these vaccines undergoing clinical evaluation followed by implementation of different vaccination strategies (Kaur and Gupta, 2020; Liu et al., 2022). A vaccine is a biologic that exposes and trains the body's immune system so that it may fight a disease it has not encountered before (Du et al., 2022). The development and widespread use of an effective SARS-CoV-2 vaccine could prevent substantial morbidity and mortality associated with COVID-19 and mitigate the secondary effects of non-pharmaceutical interventions (Shattock et al., 2022).

While collaborating with pharmaceutical giants and medical start-ups, the scientific community has successfully repurposed drugs and developed vaccines and devices to impede the progress of this overwhelming pandemic across continents (Bharati et al., 2022; Mølhave et al., 2022; Rahmah et al., 2022). Although this has led to the identification of various COVID-19 vaccine candidates, the high and growing demand for the vaccine has become a challenge (Bubar et al., 2021). Among these candidates, the vaccines developed by Pfizer-BioNTech, Moderna COVID-19, Oxford-AstraZeneca COVID-19, Sinovac COVID-19, and Johnson & Johnson COVID-19 have received Emergency Use Authorization (EUA) from regulatory bodies in the UK, Europe, USA, China, and India (Acharya et al., 2021; Kalinke et al., 2022; Mushtaq et al., 2022; Paul et al., 2022). It has been demonstrated that these vaccines can provide reasonable levels of protection against symptomatic and severe disease with two doses administered 3–4 weeks apart (Polack et al., 2020; Baden et al., 2021; Dagan et al., 2021). This means, vaccines can contribute to herd immunity, i.e., it becomes more challenging for the disease to spread if enough people are vaccinated (Muhar et al., 2022). Besides the exceptional effort and remarkable speed of development to protect individuals and reduce the spread of SARS-CoV-2, vaccine delivery has faced challenges due to supply shortages and limited distribution capacity in several countries (Burki, 2021). Based on the characteristics of COVID-19, it is essential to devise optimal vaccination allocation strategies to prioritize who should get the first available vaccinations. Thus, this will assist in reducing the number of deaths and incidence of infection. For instance, the severity and mortality of the COVID-19 disease differ by age, the number of comorbidities and gender (Jin J.-M. et al., 2020; Li X. et al., 2020). Therefore, there is a critical need to implement a vaccination priority strategy depending on these factors to mitigate the incidences and mortalities.

Outstanding efforts have been made to manufacture safe and effective COVID-19 vaccines, which many countries have already procured. However, ensuring fair and equitable worldwide access to the vaccine and subsequent roll-out faces serious challenges (Persad et al., 2020; Forman et al., 2021). Some of these challenges embrace safe and secure vaccine transportation and delivery (Wouters et al., 2021), managing fair vaccine allocation (National Academies of Sciences, 2020; Moodley and Rossouw, 2021), promoting vaccine uptake (Mello et al., 2020), ethical implications of vaccine passports and adapting clinical systems (Manisty et al., 2021; Wilf-Miron et al., 2021). Thus, the vaccine's design, development and delivery to the market to achieve national herd immunity against COVID-19 present significant policy challenges. The decision-makers must be aware of these challenges and strategize solutions at scale to address them. Considering that the COVID-19 vaccines are in limited supply (Kim et al., 2021; Mushtaq et al., 2022), the main question is how and who must get the vaccine first, i.e., which population group must have prioritized access to the COVID-19 vaccine while recognizing ethical values and achieving fairness and avoiding arbitrariness, waste, and unfair roll-out of the COVID-19 vaccine (Russell and Greenwood, 2021). We compare at the start of the pandemic the epidemiological variations in COVID-19 patients in Botswana and South Africa. This is achieved by leveraging artificial intelligence to optimize fair vaccine allocation or roll-out.

Using Botswana and Gauteng hospitalization data, we compare the population hospitalized for COVID-19 and investigate the differences in risk factors, clinical characteristics, and outcomes. A comprehensive investigation of the outcomes and mortality rate in these two countries, risk factors for hospitalization and mortality can be used to identify populations at risk, strengthen and focus specific preventive measures on these populations, and assist in defining the future needs of healthcare facilities. Moreover, it helps in learning, policymaking, and planning for future pandemics. Therefore, understanding the relationship between age, comorbidities, and health outcomes is critical in assessing and managing public health risks. As a result, the following variables were extracted for each hospitalized patient: age, sex, transfer to an intensive care unit (ICU), and hospital death. In addition, we also include all diagnoses recorded for each patient to analyze comorbidities such as tuberculosis, asthma, cardiac disease, hypertension, diabetes, HIV, obesity, chronic renal failure, and chronic pulmonary disease.

Except for this introduction, this work is arranged according to the following. First, Section 2 provides the status of the COVID-19 pandemic in Botswana, especially measures the country has taken to curb it. Moreover, the age structure of Botswana and Gauteng (South Africa) populations are compared, and Botswana's comorbidity situation is investigated. Then, in Section 3, we provide the methodology, especially details about the proposed AI model. This is followed by Section 4 on deep learning and how it is used to classify people who need vaccines based on health status characteristics. Moreover, we justify using deep neural networks and provide a comparative study. Lastly, Section 5 provides the conclusion.

## 2. COVID-19 pandemic in Botswana

After the WHO confirmed the novel coronavirus in January 2020, Botswana confirmed its first three cases in March 2020^1^. Since then, there has been a significant increase in new COVID-19 positive cases. However, while there have been recoveries, the number of deaths has continued to rise throughout the country^2^, ^3^. Botswana spends a significant amount per capita on health, enabling access to needed health services without significant severity for many citizens. Investing in the health sector has unquestionably assisted Botswana's comprehensive response to the pandemic, regardless of challenges such as shortage of health care, a deficit of intensive care facilities, ventilators, and trained health care professionals to manage the pandemic. As a response, based on the IHR2005, which is incorporated into Botswana's Public Health Act, including the Emergency Powers Act 2020, it was promulgated to address the COVID-19 pandemic. Precisely, the Government of Botswana introduced measures such as public education, isolation or advised self-quarantine, extreme social distancing, zoning strategy, community lockdown, travel restrictions interventions, and border control measures.

Moreover, the government declared a 6-month state of public emergency to take appropriate and stringent measures to address the risks presented by the COVID-19 pandemic. Notably, in response to the pandemic, on the 26th March 2021, Botswana began the COVID-19 vaccination program^4^. The vaccines that have been administered are Pfizer-BioNTech, CoronaVac, Covaxin, Janssen, Moderna, and Oxford-AstraZeneca^5^. While the vaccination program has faced several administrative challenges, it has been administered through phases based on age groups. The phase 1 vaccination program began in March 2021, and consisted of those aged 55 years and older. The vaccination of phase 2 started at the end of July 2021. While the government has made progress in vaccinating the 2.5-million population of Botswana, there has not been a clear vaccine roll-out strategy. According to literature, Botswana has some of the world's highest rates of comorbidities, increasing the severity of COVID-19 complications. Also, compared to other middle-income populations, the country has lower inpatient care facilities, thus posing additional pressure on its health system.

Several organizations have contributed and developed initiatives to curb COVID-19 transmissions. For instance, the Ministry of Health and Wellness, and Presidential Taskforce coordinate the entire health and multi-sectoral responses based on the recommendations of WHO. In addition, the WHO office in Botswana intensively supported the country's readiness and preparedness for the pandemic. Furthermore, WHO provides technical guidance and support in developing key documents such as Public Health Multi-hazard Plan, National Action Plan for Health Security, Risk Assessment tool, and National Emergency Preparedness and Response Plan and Strategy. Furthermore, it plays a crucial role in continuously reviewing other strategic instruments. In addition, the WHO facilitates engagement with other agencies of the UN, civil societies, development partners, and government sectors to find effective ways of controlling the pandemic. Additional initiatives include the Botswana-Rutgers Partnership for Health launching a series of webinars to quickly prepare the country's frontline to combat COVID-19. In addition, the training collaborated with Botswana Ministry of Health and Wellness, the University of Botswana, and the Botswana-Harvard AIDS Institute Partnership^6^.

### 2.1. Age structures of the populations of Botswana and Gauteng (South Africa)

Demographic models are still widely used in population dynamics and ecology because they are suitable for individual enumeration or census methods, and their use involves relatively simple mathematical tools.

Since it is easy to assess an individual's age, the age structure is ubiquitous in the prediction models of endemic and epidemic infections. Infectious diseases such as measles, influenza, and COVID-19 have many common characteristics; they cause frequent epidemics and have strongly age-dependent contact rates. The structure is significant for understanding and controlling these diseases. Ignoring age-structured contacts can lead to misunderstanding epidemiological data and potentially costly policy errors. In this context, and prior to vaccination, the age structure is a significant risk factor that should be considered in understanding epidemic settings (such as coronavirus pandemics) and an integral part of disease forecasting.

**Figure 1:**
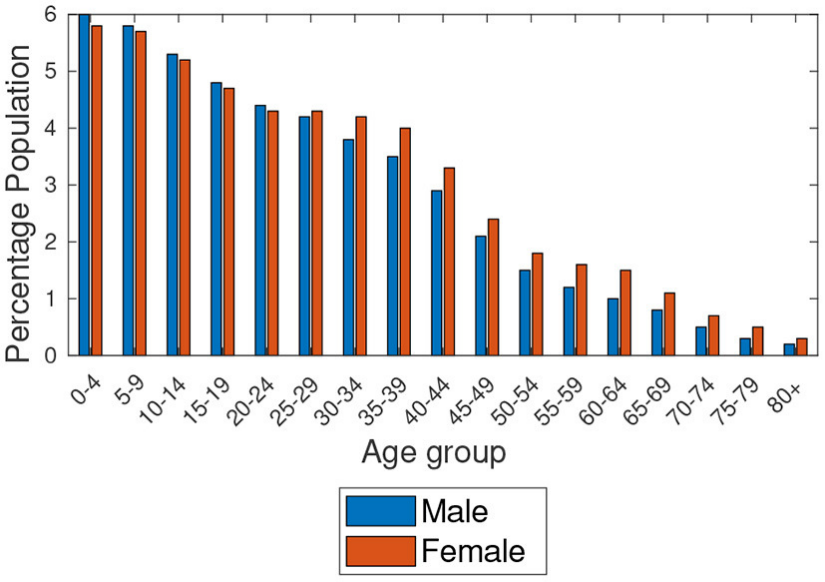
Age structures of the populations of Botswana.

**Figure 2:**
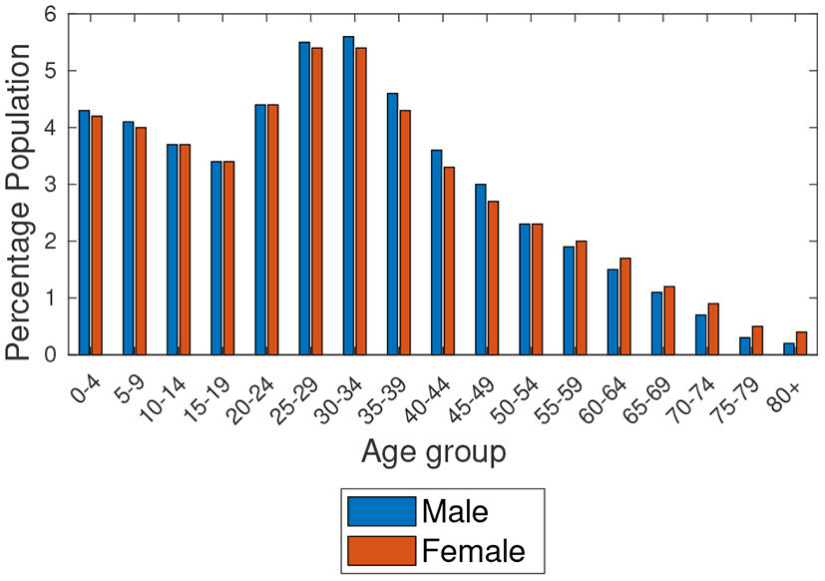
Age structures of the population's of Gauteng (South Africa).

**Figure 3:**
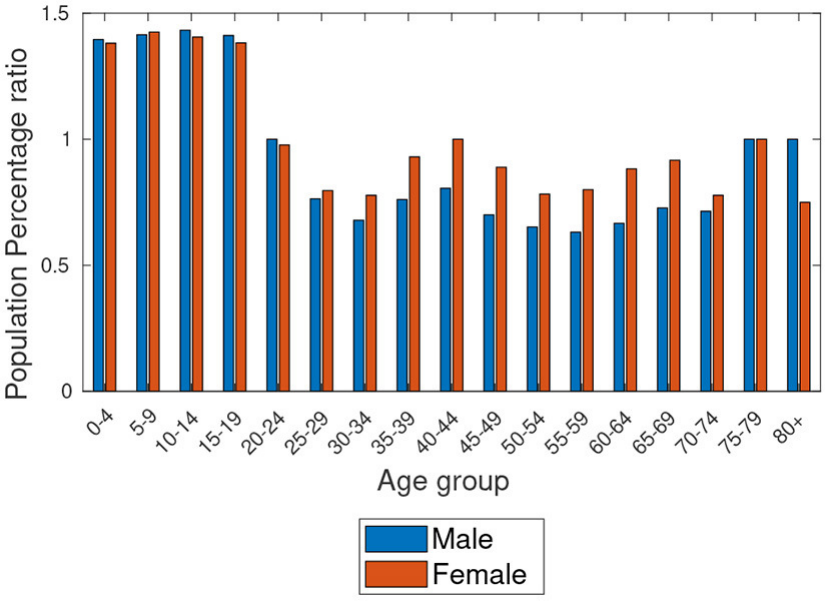
Age structures of the population's ratio of Botswana and Gauteng—South Africa.

Based on the data provided by Statistics South Africa^7^ and in Botswana (2019), Figures 1, 2 show the estimated percentage of the total population residing in Botswana and Gauteng provinces for the year 2019, respectively. The estimates show that Gauteng has the largest population compared to Botswana. Also, we observe the highest proportions of people aged 0–4 years in Botswana and 30–34 years in Gauteng, as clearly shown in the figures. Figure 3 shows the population percentage ratio of Botswana and Gauteng. Ratios are calculated by dividing Botswana's percentage over Gauteng's percentage in both males and females as given in Figures 1, 2. From Figure 3, we note that, if the *ratio*>1, in a particular age group, Botswana's proportion is higher than Gauteng's. If the *ratio* < 1, Gauteng's proportion is higher in a particular age group than in Botswana's. Moreover, if *ratio* = 1, in a particular age group, the proportion of Botswana is equal to that of Gauteng.

### 2.2. Co-morbidity situation in Botswana

Studies (Sanyaolu et al., 2020; Wang et al., 2020) suggest slow progression toward recovery amongst COVID-19 patients with underlying health conditions (or comorbidities) than their counterparts. The situation is even worse amongst the elderly population, with an increased risk of death if they contract the disease (Sanyaolu et al., 2020). Unfortunately, little is known about COVID-19 related comorbidities and prevalence rates in Botswana. Exploring such data could help understand patterns for individuals with predisposing factors and influence the COVID-19 vaccination strategy.

**Figure 4:**
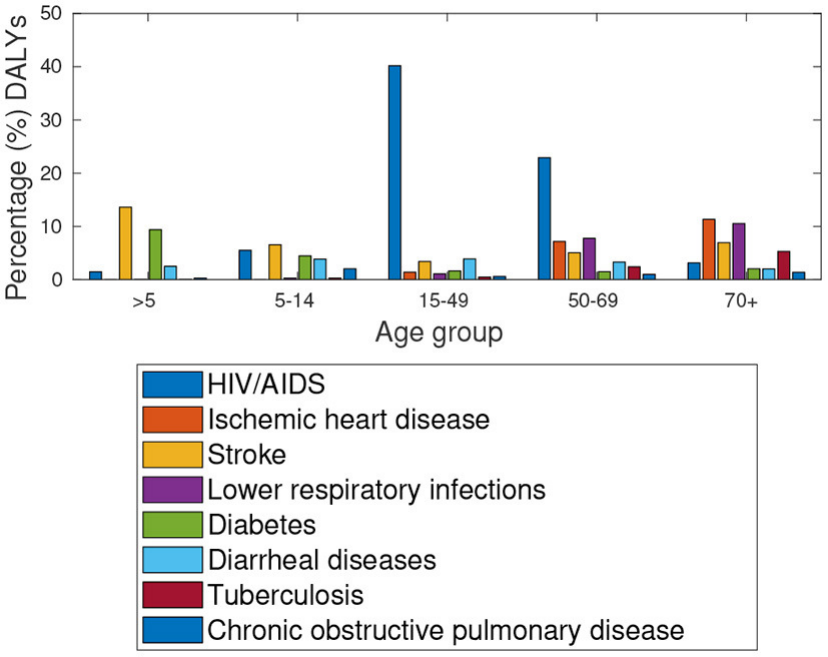
Botswana: Percentage (%) of total DALYs, Comorbidities in 2019.

**Figure 5:**
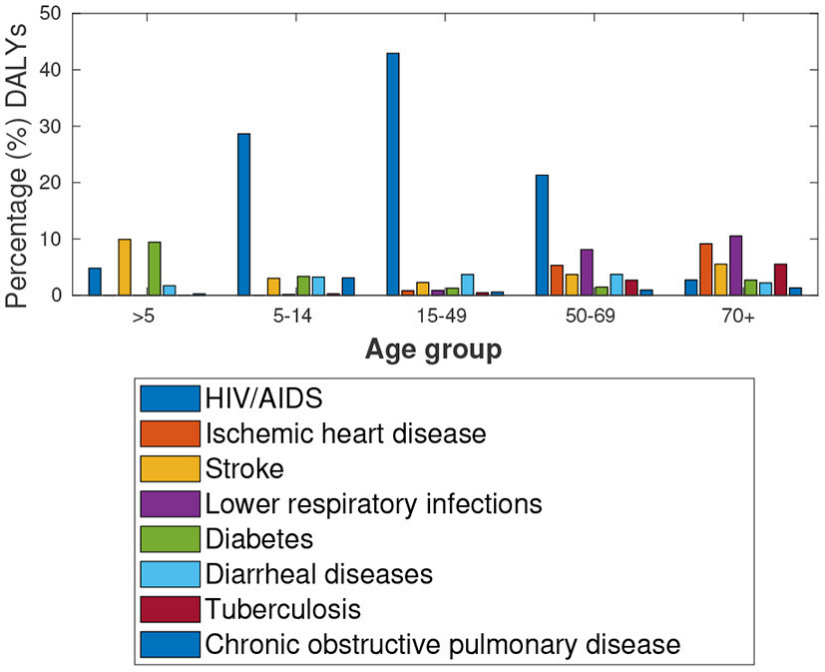
South Africa: Percentage (%) of total DALYs, Comorbidities in 2019.

The data provided by Global burden of disease study^8^ in Figure 4 suggest high HIV/AIDS prevalence rates amongst the 15–49 and 50–69 aged population group in Botswana. Notably, Figure 5 shows a similar distribution for the respective population group in South Africa. Among the older population (over 70 years), Botswana has higher incidents of lower respiratory infections, Ischaemic Heart disease and Stroke.

**Figure 6:**
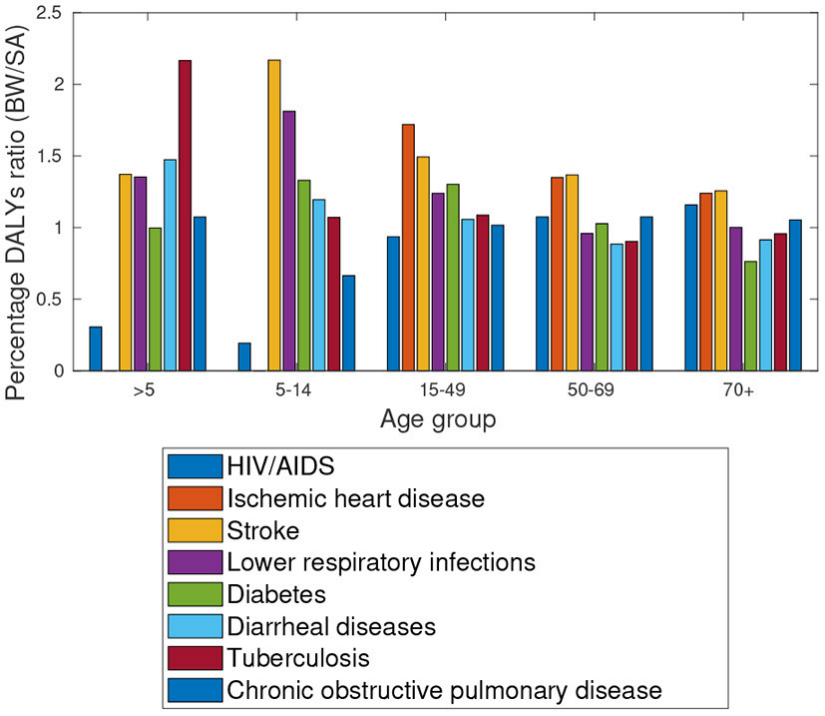
Botswana South Africa: Percentage DALYs ratio, Comorbidities in 2019.

A comparative analysis of Botswana and Gauteng data shows almost identical numbers of comorbidities (by type) amongst the older population (50–69 and 70+ age groups). Details are provided in Figure 6.

**Figure 7:**
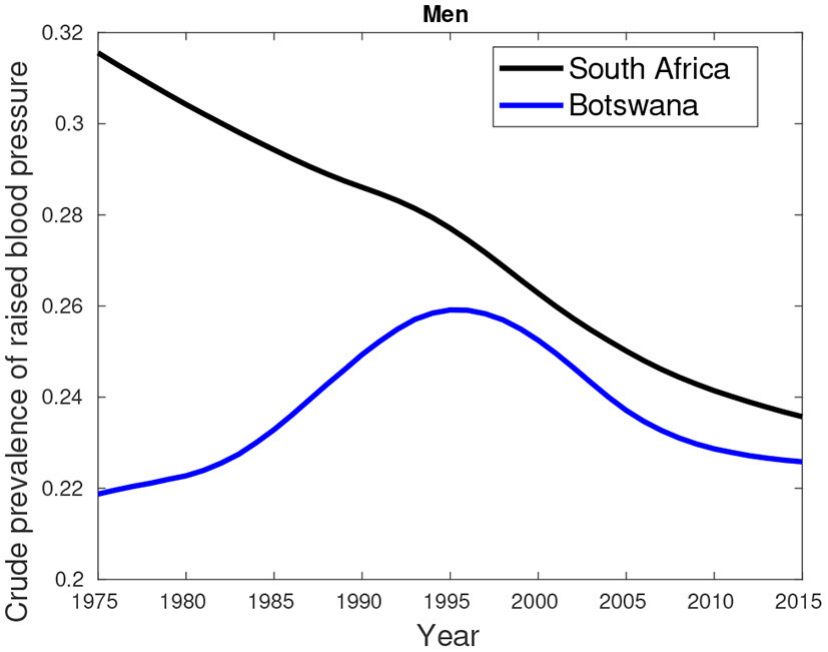
Crude prevalence of raised blood pressure for male trends in Botswana and South Africa from 1975 to 2015.

**Figure 8:**
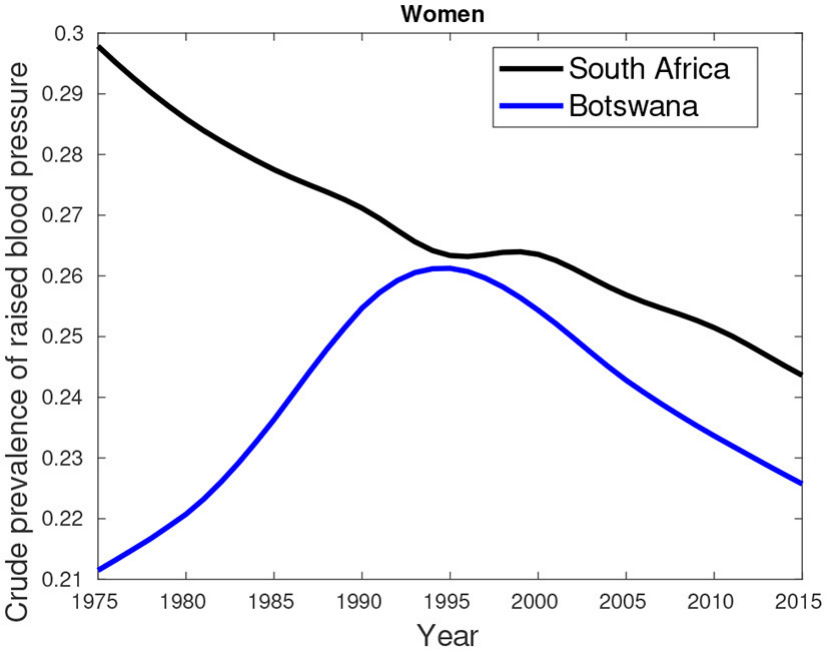
Crude prevalence of raised blood pressure for female trends in Botswana and South Africa from 1975 to 2015.

**Figure 9:**
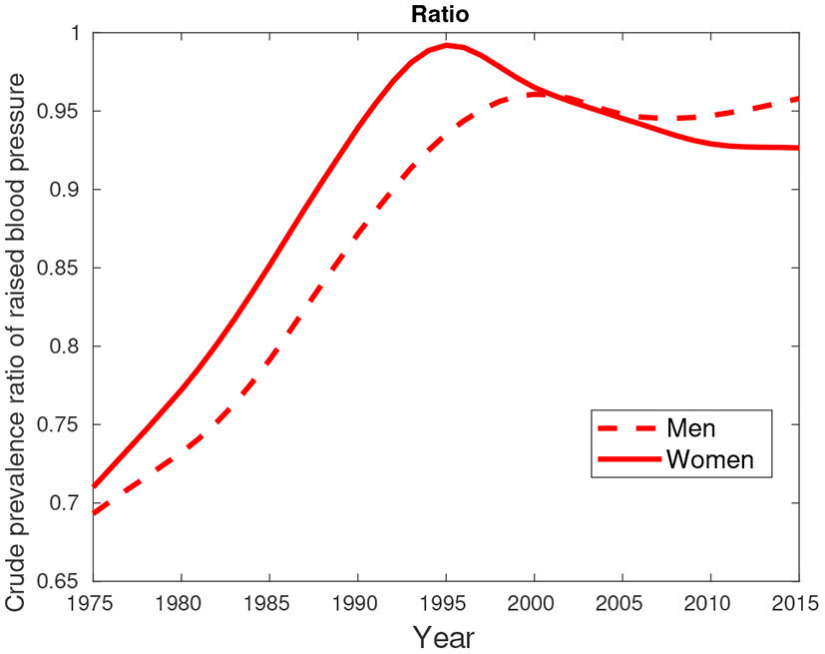
Crude prevalence of raised blood pressure for male and female ratios between Botswana and South Africa from 1975 to 2015.

An additional data about hypertension/high blood pressure (key comorbidity) excluded in the previous analyses is provided by Figures 7–9. Figures 7, 8 show higher percentages of South-Africans with hypertension compared to Botswana. The latter is true in both male and female populations. However, the difference recorded between the years 2000 and 2015 was not significant compared to the 1975–1995 period.

Figure 9 suggest hypertension was predominantly high in females compared their male counterparts. This pattern was observed in the period between 1975 and 2000. From 2005, a greater proportion of males had high blood pressure compared to females.

## 3. Methodology

Machine learning (ML) is a computational system that utilizes algorithmic and statistical models to solve problems by discovering informative hidden patterns within the data set. ML can be implemented to understand the health status of different communities within a particular country and how the population reacts to virus outbreaks. Viruses such as COVID-19 are life-threatening, and we must find measures to preserve the life-span of the population through reaching herd immunity to the virus. This can be achieved by identifying population groups that can benefit from vaccination. Through a comprehensive analysis, a deep neural network (DNN) model is chosen as the optimum classifier for different target groups. In our study, we adapt the model used in Bruce et al. (2021) which is a 14-dimensional DNN algorithm that classifies patients based on their COVID-19 health status. A multi-dimensional data set was provided by the policymakers of Botswana and South Africa to analyze the characteristics associated with severe illness. The 14 features include ethnicity, gender, age, and 11 comorbidities, which are expected to provide more information regarding the type of ward that they were admitted to and whether the patient was discharged alive or died during their prolonged hospitalization. The comorbidities found to be relevant to COVID-19 include diabetes, hypertension, asthma, cardiac disease, chronic pulmonary disease, chronic renal failure, HIV, malignancy, obesity, and tuberculosis. A DNN is trained to separate the population into two categories; the severe class, which includes patients who were hospitalized in the Intensive Care Unit, High Care, and patients who suffered mortality related to COVID-19 across all hospital wards, and the less severe class, which includes patients who were discharged alive and those who were hospitalized in the general ward.

## 4. Deep learning

The ability to automate an analytical model through ML provides a chapter on predictive models. ML is the current leader of modern society through various applications such as filtering web search content and transcribing speech into text, to mention a few. All these ML applications require deep learning to be successful and work at their optimum. It's application can be seen when combining multiple layers of neural networks leading to thorough learning of the training data set. This leads to what we refer to as deep neural networks. DNNs are considered feed forward networks due to the flow of data from the input layer to the output layer without changing direction and the connections between the layers are one way. Back propagation is used to utilize the aspect of labeling the data set to obtain the output results. Since we are dealing with a 14-dimensional problem, we should use a smart algorithm that uses deep learning to identify people who will enable herd immunity quickly through vaccines based on the characteristics of their health status. The comorbidities together with age, gender, and ethnicity are the algorithm's input variables and are used to train the model. Distinctive hyper-parameters were set together with the number of hidden layers and activation functions to yield high-efficiency results. Since we have adopted a model used in Bruce et al. (2021), we are most likely to find the same behavioral characteristics when contracting the virus, such as the two significant comorbidities enhancing COVID-19. However, the key goal would be the mortality rates between South Africa and Botswana. This is the base of our comparative study to see if the mortality in Southern Africa is similar.

### 4.1. Comparative study

**Figure 10:**
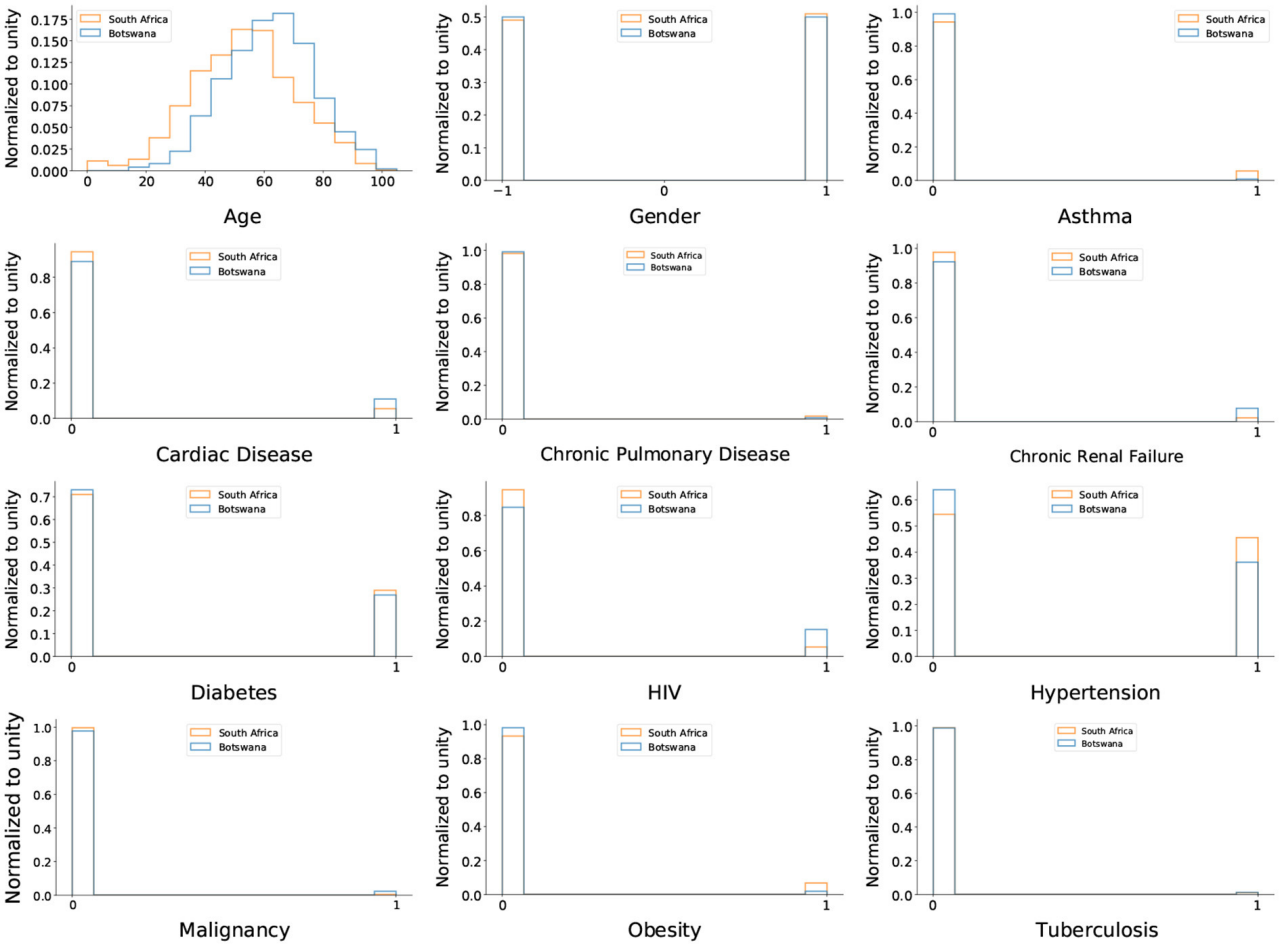
The distributions of the input variables are used to train the DNN. The blue histogram represents Botswana data, while the orange histogram represents South African data. For histograms related to comorbidities, the peak at zero denotes patients who do not have a comorbidity, while the peak at one denotes patients with comorbidity.

To prioritize and identify target groups who will benefit from vaccination, we use identical input variables as in Bruce et al. (2021), which include 11 comorbidities, age, gender, and ethnic group. To compare the health status of South Africa's population against that of Botswana, we show in Figure 10 the distributions of South African data against that of Botswana. From the distributions of the input variables used to train the model, the comorbidities of both countries are similar, thus showing that the health status of South Africa is comparable to that of Botswana. In addition, the major comorbidities that increase the severity of COVID-19 are similar for both countries: hypertension and diabetes. With that said, we anticipate that the target groups identified for vaccines in South Africa will be similar to those in Botswana since the health status of the two countries display similar characteristics.

Now that we have seen the major comorbidities that enhance the virus in both countries are the same, the first target group to receive vaccination must have both of these comorbidities. In that way, the roll out strategy will continue to be the same as the one outlined in Bruce et al. (2021). That is, the more severe group would comprise mainly elderly people, and they are the ones who need to receive vaccines in order to reduce the severity of the virus while prolonging their lives. Therefore, what is left for us is to confirm the severity of the virus by comparing the mortality rates of the virus in both countries.

#### 4.1.1. Mortality analysis

**Figure 11:**
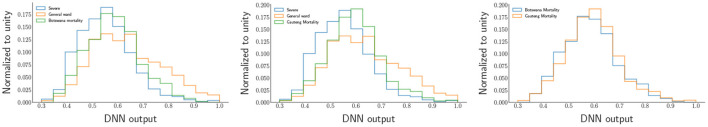
The DNN output distributions for Mortality behind COVID-19. The distribution on the left represents the mortality of Botswana, and the one on the right represents the mortality of Gauteng (South Africa).

**Figure 12:**
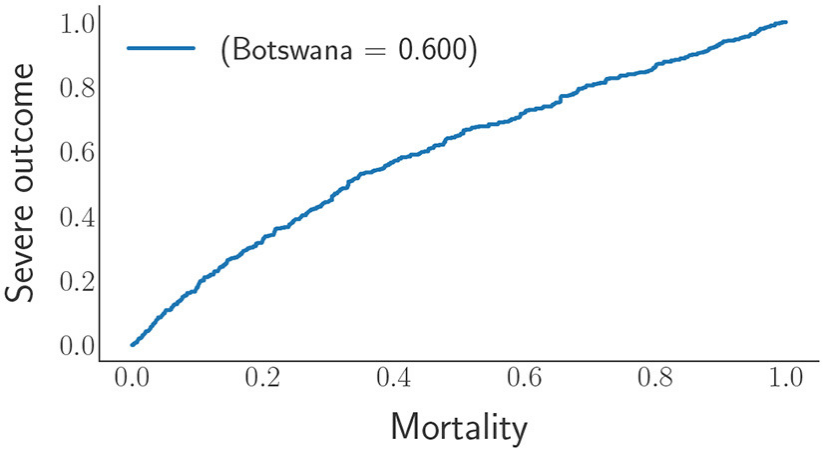
The receiver operating characteristic curve of the DNN model.

After comparing the two countries' comorbidities, we have decided to use the mortality data for both countries and input them into the DNN to see the model's performance in classifying between severe and least severe classes. Mortality needs to be classified as the most severe class. As seen, the distributions of the DNN outputs in Figure 11 demonstrate that the mortality distributions are similar to the severe class shape-wise relative to the general ward as expected. A ROC (receiver operating characteristic) curve projected by the DNN on the mortality data of Botswana is shown in Figure 12.

**Figure 13:**
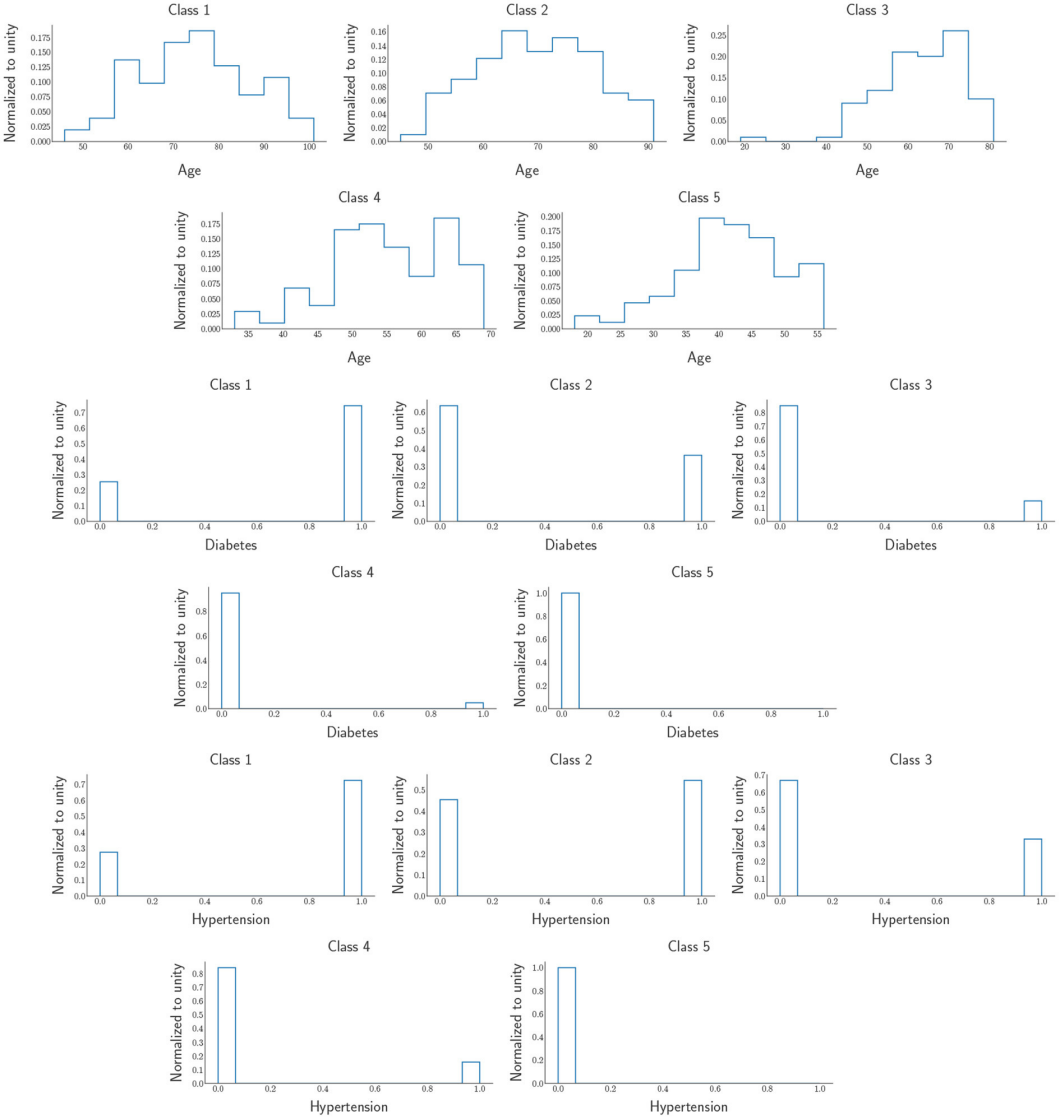
Five classes were obtained from DNN output distributions. The distributions include Age, Diabetes, and Hypertension. The first (last) row is the most (least) severe target group which is Class 1 (Class 5).

From the ROC's AUC (area under the curve), which is above 50%, we observe less discrimination between the mortality experienced due to COVID-19 in Botswana and South Africa. This shows that the status of COVID-19 in Southern African countries would undoubtedly encounter the same tragedy. According to Bruce et al. (2021), the DNN provide insights into target groups that should be vaccinated first due to the limited supply of vaccines (at the time of writing). Since we found the comorbidities critical to COVID-19, the mortality population was split into five classes, the first class (class 1) being the most severe group. In contrast, the last class (class 5) is the least severe group of the virus. Therefore, the classes have equal sizes, each counting for 20% of the area under the green graph in the DNN output distribution in Figure 11 for Botswana mortality. The distributions of the five classes were obtained from the DNN and chose three input variables to categorize the classes that need vaccines over the rest of the groups. The distributions are shown in Figure 13.

and we observe that the people that need to be prioritized when it comes to the distributions of vaccines should be the elderly as we can see from the age histograms of the five classes, more especially class 1, most of the people in this class are above 60 years of age and over 70% of them have the major comorbidities that are increasing the severity of COVID-19. As we move from class 1 to class 5, we notice that from class 2 until the last one, we can see that the people in those age regions do not have Hypertension and Diabetes. In contrast, while class 5 comprises mostly young adults; about < 1% of them have the two significant comorbidities that make COVID-19 life-threatening.

## 5. Conclusion

We compared the COVID-19 epidemic in Botswana and the Gauteng Province (South Africa). Though the population of Gauteng is more significant than Botswana's, its data is almost similar to the numbers recorded in Gauteng province, thus justifying making a reasonable comparison. Using various AI techniques, we identified several determinants such as comorbidities, including epidemiological factors on the severity of the COVID-19 pandemic. To understand the COVID-19-related comorbidities, epidemiological data, and ways to control the COVID-19 pandemic and prevalence rates, we compare the age structure of the populations between the two regions. Using various AI techniques, we find that the combination of age and comorbidities impact the severity of the COVID-19 pandemic. Notably, our model suggests that the mortality rates are similar in both countries and could be the case for the rest of the African countries since they have similar epidemiological factors. Finally, our model allows us to classify people who need vaccines based on their health status characteristics, thus allowing us to develop an appropriate vaccination strategy that complements various CPH strategies and reduces the severity of the COVID-19 pandemic in Southern Africa.

## Data availability statement

The original contributions presented in the study are included in the article/supplementary material, further inquiries can be directed to the corresponding author.

## Author contributions

All authors listed have made a substantial, direct, and intellectual contribution to the work and approved it for publication.

## Conflict of interest

The authors declare that the research was conducted in the absence of any commercial or financial relationships that could be construed as a potential conflict of interest.

## Publisher's note

All claims expressed in this article are solely those of the authors and do not necessarily represent those of their affiliated organizations, or those of the publisher, the editors and the reviewers. Any product that may be evaluated in this article, or claim that may be made by its manufacturer, is not guaranteed or endorsed by the publisher.

## Footnotes

^1^Botswana records first 3 cases of coronavirus—Health Minister. Available online at: https://www.reuters.com/article/health-coronavirus-botswana/botswana-records-first-3-cases-of-coronavirus-health-minister-idUSL8N2BK5AO (accessed September 19, 2021).

^2^Botswana: Coronavirus Pandemic Country Profile. Available online at: https://ourworldindata.org/coronavirus/country/botswana (accessed September 19, 2021).

^3^Botswana Coronavirus Cases. Available online at: https://www.worldometers.info/coronavirus/country/botswana/ (accessed September 19, 2021).

^4^About COVID-19. Available online at: https://www.gov.bw/about-covid-19 (accessed September 20, 2021).

^5^Botswana to use AstraZeneca COVID-19 vaccine. Available online at: https://news.trust.org/item/20210215154035-3mjwo/ (accessed September 20, 2021).

^6^COVID-19 in Botswana. Available online at: https://globalhealth.rutgers.edu/where-we-work/botswana/covid-19-in-botswana/ (accessed September 19, 2021).

^7^Mid-year population estimates. Available online at: https://www.statssa.gov.za/publications/P0302/P03022019.pdf (accessed June 3, 2021).

^8^GBD Compare | Viz Hub. Available online at: https://vizhub.healthdata.org/gbd-compare (accessed March 17, 2020).

## References

[B1] Acharya K. P. Ghimire T. R. Subramanya S. H. (2021). Access to and equitable distribution of COVID-19 vaccine in low-income countries. NPJ Vaccines 6, 1–3. 10.1038/s41541-021-00323-6 33854072 PMC8047027

[B2] Ahn D.-G. Shin H.-J. Kim M.-H. Lee S. Kim H.-S. Myoung J. . (2020). Current status of epidemiology, diagnosis, therapeutics, and vaccines for novel coronavirus disease 2019 (COVID-19). J. Microbiol. Biotechnol. 30, 313–324. 10.4014/jmb.2003.03011 32238757 PMC9728410

[B3] Anderson S. C. Edwards A. M. Yerlanov M. Mulberry N. Stockdale J. Iyaniwura S. A. . (2020). Genomic epidemiology of the first two waves of SARS-CoV-2 in Canada. Epidemics 35:100453. 10.7554/eLife.73896 35916373 PMC9345601

[B4] Baden L. R. El Sahly H. M. Essink B. Kotloff K. Frey S. Novak R. . (2021). Efficacy of the mRNA-1273 SARS-CoV-2 vaccine at completion of blinded phase. N. Engl. J. Med. 384, 403–416. 10.1056/NEJMoa2113017 34551225 PMC8482810

[B5] Bharati S. Podder P. Mondal M. R. H. Podder P. Kose U. (2022). A review on epidemiology, genomic characteristics, spread, and treatments of COVID-19, in Data Science for COVID-19, eds U. Kose, D. Gupta, V. H. C. de Albuquerque, and A. Khanna (Academic Press), 487–505. 10.1016/B978-0-323-90769-9.00011-6

[B6] Botswana (2019). Available online at: https://www.populationpyramid.net/botswana/2019/ (accessed June 3, 2021).

[B7] Bruce M. Wu J. Kong J. D. Bragazzi N. L. Asgary A. Kawonga M. . (2021). Leveraging artificial intelligence and big data to optimize COVID-19 clinical public health and vaccination roll-out strategies in Africa. Int. J. Environ. Res. Publ. Health 18:7890. 10.3390/ijerph18157890 34360183 PMC8345600

[B8] Bubar K. M. Reinholt K. Kissler S. M. Lipsitch M. Cobey S. Grad Y. H. . (2021). Model-informed covid-19 vaccine prioritization strategies by age and serostatus. Science 371, 916–921. 10.1126/science.abe6959 33479118 PMC7963218

[B9] Burki T. K. (2021). Challenges in the rollout of covid-19 vaccines worldwide. Lancet Respir. Med. 9, e42–e43. 10.1016/S2213-2600(21)00129-6 33684355 PMC8009608

[B10] Dagan N. Barda N. Kepten E. Miron O. Perchik S. Katz M. A. . (2021). BNT162B2 mRNA COVID-19 vaccine in a nationwide mass vaccination setting. New Engl. J. Med. 384, 1412–1423 10.1056/NEJMoa2101765 33626250 PMC7944975

[B11] Dong E. Du H. Gardner L. (2020). An interactive web-based dashboard to track COVID-19 in real time. Lancet Infect. Dis. 20, 533–534. 10.1016/S1473-3099(20)30120-1 32087114 PMC7159018

[B12] Du Z. Wang L. Pandey A. Lim W. W. Chinazzi M. Lau E. H. . (2022). Modeling comparative cost-effectiveness of SARS-CoV-2 vaccine dose fractionation in India. Nat. Med. 28 934–938. 10.1038/s41591-022-01736-z 35210596 PMC9117137

[B13] Fagbule O. (2019). 2019 novel coronavirus. Ann. Ibadan Postgrad. Med. 17, 108–110. PMC7358814 32669985

[B14] Forman R. Shah S. Jeurissen P. Jit M. Mossialos E. (2021). COVID-19 vaccine brand hesitancy and other challenges to vaccination in the Philippines. Health Policy 125, 553–567. 10.1371/journal.pgph.0000165 33820678 PMC7997052

[B15] Jin J.-M. Bai P. He W. Wu F. Liu X.-F. Han D.-M. . (2020). Gender differences in patients with covid-19: focus on severity and mortality. Front. Publ. Health 8:152. 10.3389/fpubh.2020.00152 32411652 PMC7201103

[B16] Jin Y.-H. Cai L. Cheng Z.-S. Cheng H. Deng T. Fan Y.-P. . (2020). A rapid advice guideline for the diagnosis and treatment of 2019 novel coronavirus (2019-nCoV) infected pneumonia (standard version). Milit. Med. Res. 7, 1–23. 10.1186/s40779-020-0233-6 32029004 PMC7003341

[B17] Kalinke U. Barouch D. H. Rizzi R. Lagkadinou E. Türeci Ö. Pather S. . (2022). Clinical development and approval of COVID-19 vaccines. Expert Rev. Vaccines 21, 609–619. 10.1080/14760584.2022.2042257 35157542 PMC8935460

[B18] Kaur S. P. Gupta V. (2020). COVID-19 vaccine: a comprehensive status report. Virus Res. 288:198114. 10.1016/j.virusres.2020.198114 32800805 PMC7423510

[B19] Kim J. H. Marks F. Clemens J. D. (2021). Looking beyond COVID-19 vaccine phase 3 trials. Nat. Med. 27, 205–211. 10.1038/s41591-021-01230-y 33469205

[B20] Le T. T. Andreadakis Z. Kumar A. Román R. G. Tollefsen S. Saville M. . (2020). The covid-19 vaccine development landscape. Nat. Rev. Drug Discov. 19, 305–306. 10.1038/d41573-020-00151-8 32273591

[B21] Li Q. Guan X. Wu P. Wang X. Zhou L. Tong Y. . (2020). Early transmission dynamics in wuhan, china, of novel coronavirus-infected pneumonia. New Engl. J. Med. 382, 1199–1207 10.1056/NEJMoa2001316 31995857 PMC7121484

[B22] Li X. Xu S. Yu M. Wang K. Tao Y. Zhou Y. . (2020). Risk factors for severity and mortality in adult COVID-19 inpatients in Wuhan. J. Allergy Clin. Immunol. 146, 110–118. 10.1016/j.jaci.2020.04.006 32294485 PMC7152876

[B23] Liu Y. Liu J. Shi P.-Y. (2022). SARS-CoV-2 variants and vaccination. Zoonoses 2:6. 10.15212/ZOONOSES-2022-0001 35284912 PMC8909890

[B24] Manisty C. Otter A. D. Treibel T. A. McKnight Á. Altmann D. M. Brooks T. . (2021). Antibody response to first bnt162b2 dose in previously SARS-CoV-2-infected individuals. Lancet 397, 1057–1058. 10.1016/S0140-6736(21)00501-8 33640038 PMC7972310

[B25] Mello M. M. Silverman R. D. Omer S. B. (2020). Ensuring uptake of vaccines against SARS-CoV-2. N. Engl. J. Med. 383, 1296–1299. 10.1056/NEJMp2020926 32589371

[B26] Mølhave M. Agergaard J. Wejse C. (2022). Clinical management of COVID-19 patients-an update, in Seminars in Nuclear Medicine, Vol. 52 (Elsevier), 4–10. 10.1053/j.semnuclmed.2021.06.004 PMC8206588 34243904

[B27] Moodley K. Rossouw T. (2021). What could fair allocation of an efficacious COVID-19 vaccine look like in South Africa? Lancet Glob. Health 9, e106–e107. 10.1016/S2214-109X(20)30474-5 33160456 PMC7831724

[B28] Muhar B. K. Nehira J. Malhotra A. Kotchoni S. O. (2022). The race for COVID-19 vaccines: the various types and their strengths and weaknesses. J. Pharm. Pract. 10.1177/08971900221097248. [Epub ahead of print]. 35723017 PMC9207585

[B29] Mushtaq H. A. Khedr A. Koritala T. Bartlett B. N. Jain N. K. Khan S. A. (2022). A review of adverse effects of COVID-19 vaccines. Le Infezioni Med. 30:1. 10.53854/liim-3001-1 35350266 PMC8929726

[B30] National Academies of Sciences (2020). Social Isolation and Loneliness in Older Adults: Opportunities for the Health Care System. National Academies Press. 32510896

[B31] Paul P. Janjua E. AlSubaie M. Ramadorai V. Mushannen B. Vattoth A. L. . (2022). Anaphylaxis and related events post-COVID-19 vaccination: a systematic review. J. Clin. Pharmacol. 10.1002/jcph.2120. [Epub ahead of print]. 35794852 PMC9349886

[B32] Persad G. Peek M. E. Emanuel E. J. (2020). Fairly prioritizing groups for access to COVID-19 vaccines. JAMA 324, 1601–1602. 10.1001/jama.2020.18513 32910182

[B33] Polack F. P. Thomas S. J. Kitchin N. Absalon J. Gurtman A. Lockhart S. . (2020). Safety and efficacy of the BNT162b2 mRNA COVID-19 vaccine. N. Engl. J. Med. 383 2603–2615. 10.1056/NEJMoa2034577 33301246 PMC7745181

[B34] Rahmah L. Abarikwu S. O. Arero A. G. Jibril A. T. Fal A. Flisiak R. . (2022). Oral antiviral treatments for covid-19: opportunities and challenges. Pharmacol. Rep. 1–24. 10.1007/s43440-022-00388-7 35871712 PMC9309032

[B35] Rothan H. A. Byrareddy S. N. (2020). The epidemiology and pathogenesis of coronavirus disease (COVID-19) outbreak. J. Autoimmun. 109:102433. 10.1016/j.jaut.2020.102433 32113704 PMC7127067

[B36] Russell F. M. Greenwood B. (2021). Who should be prioritised for COVID-19 vaccination? Hum. Vaccines Immunother. 17, 1317–1321. 10.1080/21645515.2020.1827882 33141000 PMC8078651

[B37] Sanyaolu A. Okorie C. Marinkovic A. Patidar R. Younis K. Desai P. . (2020). Comorbidity and its impact on patients with COVID-19. SN Comprehens. Clin. Med. 2, 1069–1076. 10.1007/s42399-020-00363-4 32838147 PMC7314621

[B38] Shattock A. J. Le Rutte E. A. Dünner R. P. Sen S. Kelly S. L. Chitnis N. . (2022). Impact of vaccination and non-pharmaceutical interventions on SARS-CoV-2 dynamics in Switzerland. Epidemics 38:100535. 10.1016/j.epidem.2021.100535 34923396 PMC8669952

[B39] Sohrabi C. Alsafi Z. O'neill N. Khan M. Kerwan A. Al-Jabir A. . (2020). World health organization declares global emergency: a review of the 2019 novel coronavirus (COVID-19). Int. J. Surg. 76, 71–76. 10.1016/j.ijsu.2020.02.034 32112977 PMC7105032

[B40] Tuite A. R. Fisman D. N. Greer A. L. (2020). Mathematical modelling of covid-19 transmission and mitigation strategies in the population of Ontario, Canada. CMAJ 192, E497–E505. 10.1503/cmaj.200476 32269018 PMC7234271

[B41] Wang Y. Lu X. Li Y. Chen H. Chen T. Su N. . (2020). Clinical course and outcomes of 344 intensive care patients with COVID-19. Am. J. Respir. Crit. Care Med. 201, 1430–1434. 10.1164/rccm.202003-0736LE 32267160 PMC7258632

[B42] Wilf-Miron R. Myers V. Saban M. (2021). Incentivizing vaccination uptake: the “green pass” proposal in Israel. JAMA 325, 1503–1504. 10.1001/jama.2021.4300 33720271

[B43] World Health Organization (2020). Mental Health and Psychosocial Considerations During the COVID-19 Outbreak. Technical report, World Health Organization.

[B44] Wouters O. J. Shadlen K. C. Salcher-Konrad M. Pollard A. J. Larson H. J. Teerawattananon Y. . (2021). Challenges in ensuring global access to COVID-19 vaccines: production, affordability, allocation, and deployment. Lancet 397 1023–1034. 10.1016/S0140-6736(21)00306-8 33587887 PMC7906643

